# Supplementary material for: High-resolution landscape of an antibiotic binding site
Source: Nature. 2023 Aug 30;622(7981):180–7. doi: 10.1038/s41586-023-06495-6 (PMC10550828; doi:10.1038/s41586-023-06495-6)
Supplement: Supplementary file 1 — Supplementary Figs. 1 and 2, containing the gating strategy for Fig. 3 and volcano plots for RNAseq data related to Extended Data Fig. 8, and Supplementary Tables 1–5, which list the strains and primers used in the study. [file 41586_2023_6495_MOESM1_ESM.pdf]

nature portfolio

https://doi.org/10.1038/s41586-023-06495-6

Supplementary information

High-resolution landscape of an antibiotic binding site

In the format provided by the authors and unedited

a

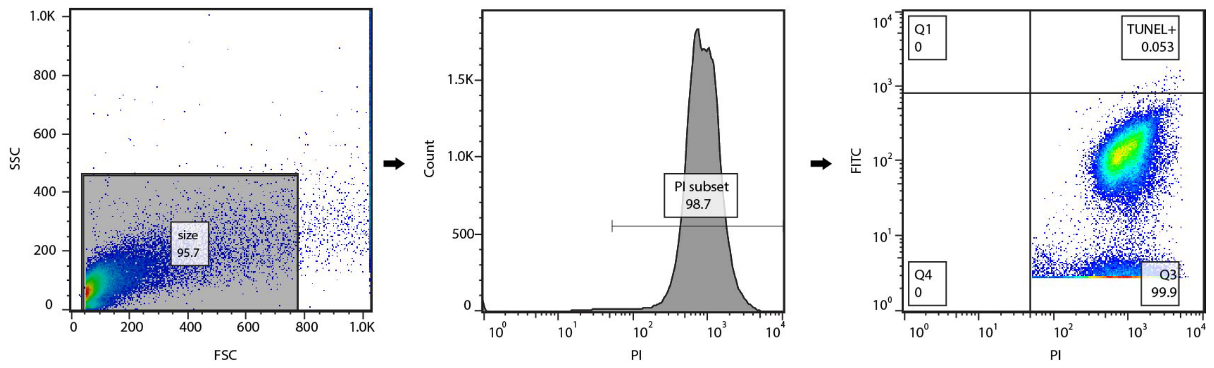

Supplementary Fig. 1. Gating strategy for Fig. 3g. a, Plots are from representative untreated sample of wild-type cells grown in LB, gating such that TUNEL-positive cells exceed signal detected in >99% of untreated cells.

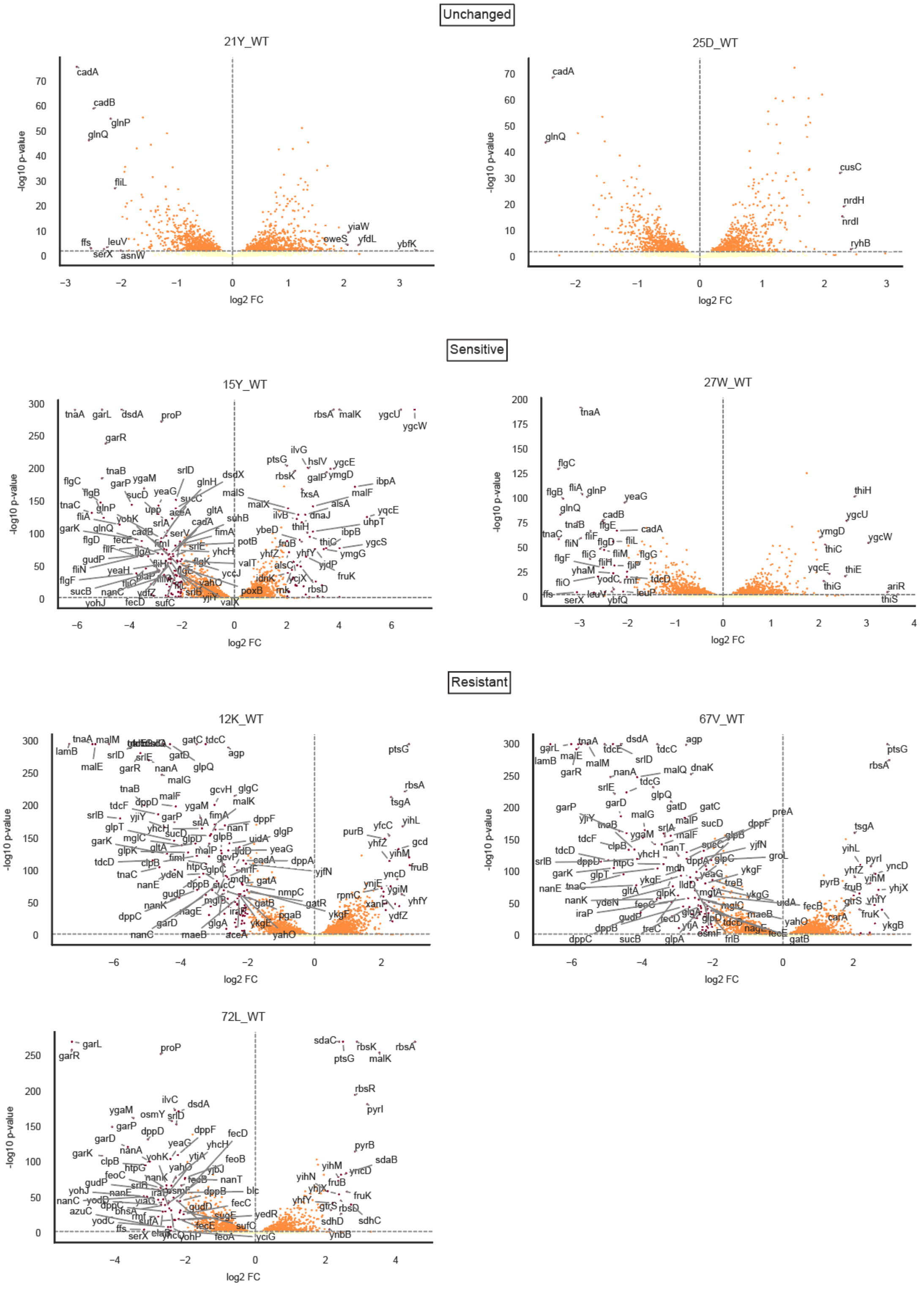

Supplementary Fig. 2. Volcano plots for RNAseq data related to Extended Data Fig. 8. Volcano plots displaying differential gene expression of each *rpoB* mutant compared to wild-type *E. coli*. Genes are labeled above and below an absolute log2 fold change of 2. RNAseq was performed in n=3 biologically independent replicates. Two tailed p values are calculated using the Wald test corrected for multiple testing using the Benjamini-Hochberg method.

Supplementary Table 1. Strains used in this study

***E. coli* strains**	**Description**	**Source or reference**
EcNR2, *E. coli*: strain MG1655, mutS$^-$, λ-Red$^+$	MAGE strain, temperature sensitive	Addgene 26931
MG1655	Wild-type	Lab stock
MG1655 *rpoB* 521Y, *rpoC*::kn	Rifampicin hypersensitive	This work
MG1655 *rpoB* 525D, *rpoC*::kn	Rifampicin hypersensitive	This work
MG1655 *recBCD*::cm	DNA repair impaired	This work
MG1655 *rpoB* 521Y, *rpoC*::kn *recBCD*::cm	Rifampicin hypersensitive, DNA repair impaired	This work
MG1655 *rpoB* 525D, *rpoC*::kn *recBCD*::cm	Rifampicin hypersensitive, DNA repair impaired	This work
MG1655 *recA*::cm	DNA repair impaired	This work
MG1655 *rpoB* 521Y, *rpoC*::kn *recA*::cm	Rifampicin hypersensitive, DNA repair impaired	This work
MG1655 *rpoB* 525D, *rpoC*::kn *recA*::cm	Rifampicin hypersensitive, DNA repair impaired	This work
MG1655 *rpoB* 515G, *rpoC*::kn	5FU, BCM sensitive	This work
MG1655 *rpoB* 515Y, *rpoC*::kn	5FU, BCM sensitive	This work
MG1655 *rpoB* 523P, *rpoC*::kn	5FU, BCM sensitive	This work
MG1655 *rpoB* 523W, *rpoC*::kn	5FU, BCM sensitive	This work
MG1655 *rpoB* 526V, *rpoC*::kn	5FU, BCM sensitive	This work
MG1655 *rpoB* 527W, *rpoC*::kn	5FU, BCM sensitive	This work
MG1655 *rpoB* 512K, *rpoC*::kn	5FU, BCM resistant	This work
MG1655 *rpoB* 567C, *rpoC*::kn	5FU, BCM resistant	This work
MG1655 *rpoB* 567V, *rpoC*::kn	5FU, BCM resistant	This work
MG1655 *rpoB* 572L, *rpoC*::kn	5FU, BCM resistant	This work
MG1655 *thyA*::tet	Thymidine auxotrophy	[68]
MG1655 *rpoC*::kn, *thyA*::tet	Thymidine auxotrophy	This work
MG1655 *rpoB* 515Y, *rpoC*::kn, *thyA*::tet	Thymidine auxotrophy	This work
MG1655 *rpoB* 526V, *rpoC*::kn, *thyA*::tet	Thymidine auxotrophy	This work
MG1655 *rpoB* 527W, *rpoC*::kn, *thyA*::tet	Thymidine auxotrophy	This work
MG1655 *rpoC*::kn, *pyrD*	pyrD deletion	This work
MG1655 *rpoB* 515Y, *rpoC*::kn, *pyrD*	pyrD deletion	This work
MG1655 *rpoB* 526V, *rpoC*::kn, *pyrD*	pyrD deletion	This work
MG1655 *rpoB* 527W, *rpoC*::kn, *pyrD*	pyrD deletion	This work
MG1655 *rpoC*::kn, *upp*::cm	upp deletion	This work
MG1655 *rpoB* 515Y, *rpoC*::kn, *upp*::cm	upp deletion	This work

MG1655 *rpoB* 526V, *rpoC*::kn, *upp*::cm	upp deletion	This work
MG1655 *rpoB* 527W, *rpoC*::kn, *upp*::cm	upp deletion	This work
MG1655 *rpoB* 513P	Rifampicin resistant	This work
MG1655 *rpoB* 513P, *thyA*::tet	Rifampicin resistant, thyA deletion	This work
PC2 *rpoC*::kn	Temperature sensitive allele of dnaC2. F-, leuB6(Am), λ-, thyA47, rpsL153(strR), dnaC2(ts), dnaT12(ts), deoC3	[43]
PC2 *rpoB* 521Y, *rpoC*::kn	Temperature sensitive allele of dnaC2	This work
PC2 *rpoB* 525D, *rpoC*::kn	Temperature sensitive allele of dnaC2	This work
MG1655 *rpoC*-10X-His	His-tagged rpoC for ChIP experiments	This work
MG1655 *rpoB* 525D, *rpoC*-10X-His	His-tagged rpoC for ChIP experiments	This work

Supplementary Table 2. Primers for MAGE-seq library construction

Name	Sequence
rpoB_bin1_F	CCTACACGACGCTCTTCCGATCTNNNNNNNGCAGTGAAAGAGTTCTTCGGTTCCA
rpoB_bin1_R	GAGTTCAGACGTGTGCTCTTCCGATCTNNNNNNNTGCACGTTCACGGGTCAGA
rpoB_bin2_F	CCTACACGACGCTCTTCCGATCTNNNNNNNCGAAGTTCGAGACGTACACCCG
rpoB_bin2_R	GAGTTCAGACGTGTGCTCTTCCGATCTNNNNNNNGGAGTCTCAAGGAAGCCGTATTCG

Supplementary Table 3. Primers for transcription kinetics RT-qPCR

Name	Sequence
lacZ P297-F	CAGCCTGAATGGCGAATGG
lacZ P297-R	CGACGACAGTATCGGCCTC
lacZ P898-F	CGTGACTACCTACGGGTAA
lacZ P898-R	GCATAACCACCACGCTCATC
lacZ P1237-F	GATGAGCAGACGATGGTGC
lacZ P1237-R	TACCACAGCGGATGGTTCG
lacZ P1578-F	GCTGGATCAAATCTGTCGATCC
lacZ P1578-R	GGAAGGGCTGGTCTTCATCC
lacZ P1941-F	CGCTGACGGAAGCAAAACA
lacZ P1941-R	GCCCGGATAAACGGAACTG

Supplementary Table 4. Primers for SLR-qPCR

Name	Sequence
fepA reference fwd	CCTGGGACGCATATTTCAATG
fepA reference rev	CCGGTCAGGCTAAAGTTAGTG
fepA SLR promoter fwd	GTTCACTTTTCCAGATAATTTCTTTGGC
fepA SLR promoter rev	CCGGTCAGGCTAAAGTTAGTG
fepA SLR gene body fwd	AAGATCATCCGTACCATGCC
fepA SLR gene body rev	TCAGGTCATCGTTACCTTGC
ilvC reference fwd	TCTGGTCTCGATATCTCCTACG
ilvC reference rev	CCATTTTCGGTCGCTTTACG
ilvC SLR promoter fwd	GCAATCAGCACTACTGCCAG
ilvC SLR promoter rev	CCCACGTTTGTACTCTTCACG
ilvC SLR gene body fwd	TCTGGTCTCGATATCTCCTACG
ilvC SLR gene body rev	GAGAACAGATAGTTACCGTACTCAGC
oriC guide RNA	GCCGGATCCTTGTTATCCAC

Supplementary Table 5. Primers for qPCR

Name	Sequence
qumuC_f	CCAGACCAAAACGCTGGCTAAG
qumuC_r	TGATAAATCCACCACCCCACCC
qrecA_f	CTTCCGGTAAAACCACGCTGAC
qrecA_r	GCACAGCAGGTTGTCGATATCG
qrpsL_f	GAGCACTCCGTGATCCTGATCC
qrpsL_r	CTTTAACGCCGGAGCAGTCAAG
qPCR_oriC_F	GGCGGAACAGGTTGAGATTC
qPCR_oriC_R	GGATACCTGGCGGTAATCCA
qPCR_TER_F	CCCATTAGTGAGTGGCGTAG
qPCR_TER_R	GGTCTTGCTCGAATCCCTTT
