# Supplementary material for: High-resolution landscape of an antibiotic binding site
Source: Nature. 2023 Aug 30;622(7981):180–7. doi: 10.1038/s41586-023-06495-6 (PMC10550828; doi:10.1038/s41586-023-06495-6)
Supplement: Supplementary file 2 — Reporting Summary [file 41586_2023_6495_MOESM2_ESM.pdf]

nature portfolio

Corresponding author(s): Aviram Rasouly, Evgeny Nudler

Last updated by author(s): Jul 7, 2023

Reporting Summary

Nature Portfolio wishes to improve the reproducibility of the work that we publish. This form provides structure for consistency and transparency in reporting. For further information on Nature Portfolio policies, see our Editorial Policies and the Editorial Policy Checklist.

Statistics

For all statistical analyses, confirm that the following items are present in the figure legend, table legend, main text, or Methods section.

n/a	Confirmed	
☐	☒	The exact sample size (*n*) for each experimental group/condition, given as a discrete number and unit of measurement
☐	☒	A statement on whether measurements were taken from distinct samples or whether the same sample was measured repeatedly
☐	☒	The statistical test(s) used AND whether they are one- or two-sided<br>*Only common tests should be described solely by name; describe more complex techniques in the Methods section.*
☐	☒	A description of all covariates tested
☐	☒	A description of any assumptions or corrections, such as tests of normality and adjustment for multiple comparisons
☐	☒	A full description of the statistical parameters including central tendency (e.g. means) or other basic estimates (e.g. regression coefficient) AND variation (e.g. standard deviation) or associated estimates of uncertainty (e.g. confidence intervals)
☐	☒	For null hypothesis testing, the test statistic (e.g. *F*, *t*, *r*) with confidence intervals, effect sizes, degrees of freedom and *P* value noted<br>*Give P values as exact values whenever suitable.*
☒	☐	For Bayesian analysis, information on the choice of priors and Markov chain Monte Carlo settings
☒	☐	For hierarchical and complex designs, identification of the appropriate level for tests and full reporting of outcomes
☐	☒	Estimates of effect sizes (e.g. Cohen's *d*, Pearson's *r*), indicating how they were calculated

Our web collection on statistics for biologists contains articles on many of the points above.

Software and code

Policy information about availability of computer code

Data collection	Sequencing data was collected on the Novaseq 6000 (Illumina) at the NYU Genome Technology Center and an in-house NextSeq 6000 (Illumina). qPCR data was collected on a QuantStudio 7 (Applied Biosystems). Flow cytometry data was collected on a FACSCalibur (BD Biosciences).
Data analysis	Sequencing data was analyzed using CutAdapt (vl.18), Bowtie2 (v2.4.1), and deeptools2 (v3.5.l). Software adapted from https://github.com/jiminpark66/MAGEseq (Park et al., 2021) was used for MAGE-seq analysis. For plots, error bars denote 95% confidence intervals and p-values are determined using the independent t-test (two-tailed) unless otherwise noted. FlowJo (vl0.6.0) was used for flow cytometry analysis. Gel intensity was quantified using GelQuant.NET software provided by biochemlabsolutions.com. PyMOL (v2.3.3) was used for visualization.

For manuscripts utilizing custom algorithms or software that are central to the research but not yet described in published literature, software must be made available to editors and reviewers. We strongly encourage code deposition in a community repository (e.g. GitHub). See the Nature Portfolio guidelines for submitting code & software for further information.

Data

Policy information about availability of data

All manuscripts must include a data availability statement. This statement should provide the following information, where applicable:

- Accession codes, unique identifiers, or web links for publicly available datasets
- A description of any restrictions on data availability
- For clinical datasets or third party data, please ensure that the statement adheres to our policy

Sequencing data is available through the NCBI sequence read archive (SRA) under PRJNA992395. Protein Data Base accession number 5UAC was used for visualization.

Research involving human participants, their data, or biological material

Policy information about studies with human participants or human data. See also policy information about sex, gender (identity/presentation), and sexual orientation and race, ethnicity and racism.

Reporting on sex and gender	No human research participants were used in this study.
Reporting on race, ethnicity, or other socially relevant groupings	No human research participants were used in this study.
Population characteristics	No human research participants were used in this study.
Recruitment	No human research participants were used in this study.
Ethics oversight	No human research participants were used in this study.

Note that full information on the approval of the study protocol must also be provided in the manuscript.

Field-specific reporting

Please select the one below that is the best fit for your research. If you are not sure, read the appropriate sections before making your selection.

☒ Life sciences ☐ Behavioural & social sciences ☐ Ecological, evolutionary & environmental sciences

For a reference copy of the document with all sections, see nature.com/documents/nr-reporting-summary-flat.pdf

Life sciences study design

All studies must disclose on these points even when the disclosure is negative.

Sample size	No statistical methods were used to predetermine sample size. Sample sizes were determined based on previously published experiments.
Data exclusions	No data was excluded.
Replication	All experiments were performed in at least 3 biologically independent replicates with similar results. The experiments were performed independently and were reproducible.
Randomization	Randomization is not applicable as there are no groups to be allocated.
Blinding	Blinding was not applicable in this study as there are no groups to be allocated. Bias from prior knowledge described in the study is considered limited.

Reporting for specific materials, systems and methods

We require information from authors about some types of materials, experimental systems and methods used in many studies. Here, indicate whether each material, system or method listed is relevant to your study. If you are not sure if a list item applies to your research, read the appropriate section before selecting a response.

Materials & experimental systems

n/a	Involved in the study
☐	☒ Antibodies
☒	☐ Eukaryotic cell lines
☒	☐ Palaeontology and archaeology
☒	☐ Animals and other organisms
☒	☐ Clinical data
☒	☐ Dual use research of concern
☒	☐ Plants

Methods

n/a	Involved in the study
☐	☒ ChIP-seq
☐	☒ Flow cytometry
☒	☐ MRI-based neuroimaging

Antibodies

Antibodies used	E. coli RNA Polymerase beta Monoclonal Antibody clone 8RB13 mouse mAB (663905; Biolegend), Lex A Antibody (E-7) (sc-365999; Santa Cruz), 6X-His-tag Monoclonal Antibody (66005-1-Ig; Proteintech).
Validation	Antibodies were validated using western blot following manufacturer protocols with details provided in the Methods section. Antibodies used for ChIP were validated using western blot prior to performing experiments following manufacturer protocols. All antibodies have been used and cited extensively in house and in the literature.

Plants

Seed stocks	N/A
Novel plant genotypes	N/A
Authentication	N/A

ChIP-seq

Data deposition

☒ Confirm that both raw and final processed data have been deposited in a public database such as GEO.

☒ Confirm that you have deposited or provided access to graph files (e.g. BED files) for the called peaks.

Data access links<br>*May remain private before publication.*	Sequencing data is available through the NCBI sequence read archive (SRA) under PRJNA992395
Files in database submission	CHIP_WT_input_rep1.R1.fastq.gz<br>CHIP_WT_input_rep1.R2.fastq.gz<br>CHIP_WT_input_rep2.R1.fastq.gz<br>CHIP_WT_input_rep2.R2.fastq.gz<br>CHIP_WT_rep1.R1.fastq.gz<br>CHIP_WT_rep1.R2.fastq.gz<br>CHIP_WT_rep2.R1.fastq.gz<br>CHIP_WT_rep2.R2.fastq.gz<br>CHIP_25D_input_rep1.R1.fastq.gz<br>CHIP_25D_input_rep1.R2.fastq.gz<br>CHIP_25D_input_rep2.R1.fastq.gz<br>CHIP_25D_input_rep2.R2.fastq.gz<br>CHIP_25D_rep1.R1.fastq.gz<br>CHIP_25D_rep1.R2.fastq.gz<br>CHIP_25D_rep2.R1.fastq.gz<br>CHIP_25D_rep2.R2.fastq.gz
Genome browser session<br>(e.g. UCSC)	N/A

Methodology

Replicates	ChIP-seq experiments were provided in biologically independent duplicate with reproducible results as seen in Extended Data Fig. 4.
Sequencing depth	We aimed for >10M paired-end reads per sample.
Antibodies	6X-His-tag Monoclonal Antibody (66005-1-Ig; Proteintech).

Peak calling parameters	No peak calling was performed. Reads were counted within regions of interest around transcription start sites using Deeptools. See methods for more details.
Data quality	Data was reproducible between biologically independent replicates. More reads were found around transcription start sites as previously reported.
Software	CutAdapt (vl.18), Bowtie2 (v2.4.1), and deeptools2 (v3.5.l)

Flow Cytometry

Plots

Confirm that:

- [x] The axis labels state the marker and fluorochrome used (e.g. CD4-FITC).
- [x] The axis scales are clearly visible. Include numbers along axes only for bottom left plot of group (a 'group' is an analysis of identical markers).
- [x] All plots are contour plots with outliers or pseudocolor plots.
- [x] A numerical value for number of cells or percentage (with statistics) is provided.

Methodology

Sample preparation	For DNA fragmentation experiments, cells were diluted 1,000-fold from overnight cultures and grown to an OD600 of 0.2 in 2mL of media before the addition of 80ug/mL of Rif . After 1 hour of treatment, cells were washed twice with an equal volume of LB before being fixed with 4% formaldehyde for terminal deoxynucleotide transferase dUTP nick end labeling (TUN EL). Labeling of DNA fragments was performed using the Apo-Direct Kit (BD Bioscience) following manufacturer instructions.
Instrument	FACSCalibur (BD Biosciences)
Software	FlowJo (vl0.6.0)
Cell population abundance	At least 50,000 cells were collected for each experimental condition.
Gating strategy	The percentage of TUNEL positive cells for a given condition is the percentage of cells exceeding the signal detected in >99% of untreated cells.

- [x] Tick this box to confirm that a figure exemplifying the gating strategy is provided in the Supplementary Information.
